# Supplementary material for: N-Functionalization of 5-Aminotetrazoles: Balancing Energetic Performance and Molecular Stability by Introducing ADNP
Source: Int J Mol Sci. 2022 Dec 13;23(24):15841. doi: 10.3390/ijms232415841 (PMC9779898; doi:10.3390/ijms232415841)
Supplement: Supplementary file 1 [file ijms-23-15841-s001.zip › ijms-2012883-supplementary.pdf]

International Journal of
Molecular Sciences

MDPI

Article: Electronic Supplementary Information (ESI)

N-Functionalization of 5-Aminotetrazoles: Balancing Energetic Performance and Molecular Stability by Introducing ADNP

Jin Xiong [1], Jinjie Chang [1], Jinxiong Cai [1], Ping Yin [1,2,*] and Siping Pang [1,*]

1 School of Materials Science and Engineering, Beijing Institute of Technology, Beijing 100081, China
2 Beijing Institute of Technology Chongqing Innovation Center, Chongqing 401120, China
* Correspondence: pingyin@bit.edu.cn (P.Y.); pangsp@bit.edu.cn (S.P.)

Table of Contents

S1 Computational Details S2
S2 Single-crystal X-ray stuctures of compound **CDPA** S2
S3 Crystallographic Data for **DMPT-1** and **DMPT-2** S3
S4 Crystallographic Bond [Å] and Angles [°] for **DMPT-1** S3
S5 Crystallographic Bond [Å] and Angles [°] for **DMPT-2** S4
S6 ^{1}H and ^{13}C NMR spectra S5
S7 DSC curves of the title compounds S8
References S9

S1. Computational Details

The heats of formation of compounds **DMPT-1** and **DMPT-2** were performed by using the Gaussian 09 suite of programs [1-2]. Gas phase heats of formation of the title compounds were computed based on an isodesmic reaction (Scheme S1). The enthalpy of reaction was carried out by combining the M062X/6-311++G** energy difference for the reactions, the scaled zero-point energies (ZPE), values of thermal correction (HT), and other thermal factors. The solid state heats of formation were further obtained by employing Trouton's rule according to Equation (S1) (T represents either melting point or decomposition temperature when no melting occurs prior to decomposition) [3].

$$\Delta H_{sub} = 188/J\ mol^{-1}\ K^{-1} \times T \tag{S1}$$

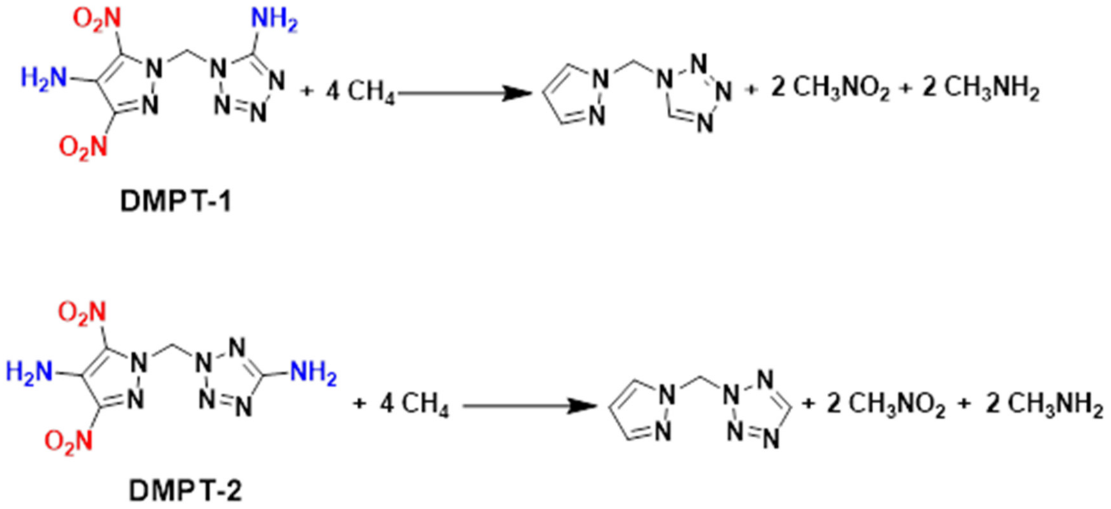

Scheme S1. Isodesmic reaction for calculating heats of formation for **DMPT-1** and **DMPT-2.**

S2. Single-crystal X-ray structures of compound CDPA.

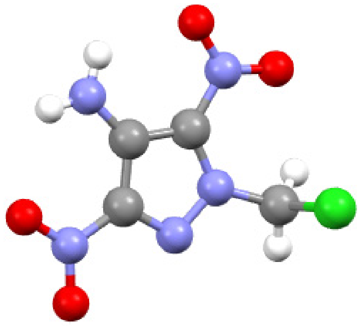

Scheme S2. Single-crystal X-ray structures of **CDPA**.

S3. Crystallographic data for DMPT-1, DMPT-2, CDPA.

Table S1. Crystal data and structure refinement for **DMPT-1, DMPT-2** and **CDPA**

**Compound**	**DMPT-1**	**DMPT-2**	**CDPA**
CCDC No.	2172948	2183788	2217429
Empirical Formula	$C_5H_6N_{10}O_4$	$C_5H_6N_{10}O_4$	$C_4H_4ClN_5O_4$
Formula Weight	270.20	270.20	221.57
Temperature (K)	296(2)	296(2)	150
Crystal System	Monoclinic	Orthorhombic	Monoclinic
Space group	*P2₁/c*	*P2₁2₁2₁*	*P2₁/c*
Unit cell dimensions			
a (Å)	16.59(2)	5.4960(8)	14.5620(11)
b (Å)	5.841(8)	11.0691(17)	7.3553(6)
c (Å)	10.755(14)	16.665(3)	7.6104(6)
α (°)	90	90	90
β (°)	107.568(17)	90	7.3553(6)
γ (°)	90	90	90
Volume (Å^3)	994(2)	1013.8(3)	801.74(11)
Z	4	4	16
Density (g cm^{-3}) (calculated)	1.806	1.770	1.836
F(000)	552.0	552.0	448.0
Crystal size (mm^3)	0.180 x 0.160 x 0.130	0.190 x0.160 x 0.150	0.150 x 0.080 x 0.050
Goodness-of-fit on F^2	1.055	0.995	1.054
Final R indexes [I>=2σ (I)]	R_1 = 0.0426 wR_2 = 0.1122	R_1 = 0.0304, wR_2 = 0.0948	R_1 = 0.0523, wR_2 = 0.1068
Final R indexes [all data]	R1 = 0.0598, wR2 = 0.1223	R_1 = 0.0318, wR_2 = 0.0963	R_1 = 0.0935, wR_2 = 0.1268

S4. Crystallographic Bond [Å] and Angles [°] for DMPT-1

Table S2. Crystallographic Bond and Angles for **DMPT-1**

Atom-Atom	Length /Å	Atom-Atom	Length /Å
O(1)-N(1)	1.228(2)	N(5)-C(12)	1.377(3)
O(2)-N(1)	1.212(2)	N(6)-N(7)	1.366(3)
O(3)-N(2)	1.244(2)	N(6)-C(4)	1.442(3)
O(4)-N(2)	1.223(2)	N(6)-C(16)	1.354(2)
N(1)-C(7)	1.434(3)	N(7)-N(8)	1.287(2)
N(2)-C(12)	1.394(3)	N(8)-N(9)	1.356(3)
N(3)-N(5)	1.330(2)	N(9)-C(16)	1.327(2)
N(3)-C(7)	1.323(3)	N(10)-C(16)	1.334(3)
N(4)-C(10)	1.332(3)	C(7)-C(10)	1.409(3)
N(5)-C(4)	1.453(2)	C(10)-C(12)	1.400(3)
**Atom-Atom-Atom**	**Angle/°**	**Atom-Atom-Atom**	**Angle/°**
O(1)-N(1)-C(7)	116.07(18)	C(16)-N(9)-N(8)	106.04(17)
O(2)-N(1)-O(1)	124.34(18)	N(6)-C(4)-N(5)	110.73(14)
O(2)-N(1)-C(7)	119.59(18)	N(3)-C(7)-N(1)	119.11(17)
O(3)-N(2)-C(12)	116.60(16)	N(3)-C(7)-C(10)	114.56(17)
O(4)-N(2)-O(3)	123.99(17)	C(10)-C(7)-N(1)	126.33(18)
O(4)-N(2)-C(12)	119.41(17)	N(4)-C(10)-C(7)	129.89(18)
C(7)-N(3)-N(5)	105.09(15)	N(4)-C(10)-C(12)	129.21(19)
N(3)-N(5)-C(4)	117.65(15)	C(12)-C(10)-C(7)	100.89(17)
N(3)-N(5)-C(12)	110.88(14)	N(2)-C(12)-C(10)	126.97(17)
C(12)-N(5)-C(4)	131.31(16)	N(5)-C(12)-N(2)	124.48(16)
N(7)-N(6)-C(4)	121.19(14)	N(5)-C(12)-C(10)	108.55(17)
C(16)-N(6)-N(7)	107.94(14)	N(9)-C(16)-N(6)	108.09(17)
C(16)-N(6)-N(7)	130.22(16)	N(9)-C(16)-N(10)	126.33(18)
N(8)-N(7)-N(6)	106.20(14)	N(10)-C(16)-N(6)	125.56(17)
N(7)-N(8)-N(9)	111.68(15)		

S5. Crystallographic Bond [Å] and Angles [°] for DMPT-2

Table S3. Crystallographic Bond and Angles for **DMPT-2**

Atom-Atom	Length /Å	Atom-Atom	Length /Å
O(1)-N(10)	1.230(2)	N(5)-C(9)	1.357(3)
O(2)-N(10)	1.231(2)	N(5)-N(7)	1.331(2)
O(3)-N(8)	1.221(3)	N(6)-C(1)	1.444(2)
O(4)-N(8)	1.225(3)	N(6)-C(4)	1.377(2)
N(1)-C(9)	1.349(3)	N(7)-C(2)	1.322(2)
N(2)-N(3)	1.340(2)	N(8)-C(2)	1.430(3)
N(2)-C(9)	1.331(3)	N(9)-C(3)	1.334(3)
N(3)-N(4)	1.306(2)	N(10)-C(4)	1.400(2)
N(3)-C(1)	1.455(3)	C(2)-C(3)	1.420(3)
N(4)-N(5)	1.313(2)	C(3)-C(4)	1.394(3)
**Atom-Atom-Atom**	**Angle/°**	**Atom-Atom-Atom**	**Angle/°**
C(9)-N(2)-N(3)	100.57(16)	O(2)-N(10)-C(4)	119.39(17)
N(2)-N(3)-C(1)	122.47(16)	N(6)-C(1)-N(3)	109.95(15)
N(4)-N(3)-N(2)	114.58(15)	N(7)-C(2)-N(8)	118.82(17)
N(4)-N(3)-C(1)	122.92(15)	N(7)-C(2)-C(3)	114.42(16)
N(3)-N(4)-N(5)	106.38(14)	C(3)-C(2)-N(8)	126.71(17)
N(4)-N(5)-C(9)	105.94(17)	N(9)-C(3)-C(2)	129.26(19)
N(7)-N(6)-C(1)	116.60(15)	N(9)-C(3)-C(4)	130.11(19)
N(7)-N(6)-C(4)	110.82(15)	C(4)-C(3)-C(2)	100.63(16)
C(4)-N(6)-C(1)	130.90(16)	N(6)-C(4)-N(10)	123.61(16)
C(2)-N(7)-N(6)	105.09(15)	N(6)-C(4)-C(3)	109.00(16)
O(3)-N(8)-O(4)	124.7(2)	C(3)-C(4)-N(10)	127.39(17)
O(3)-N(8)-C(2)	118.13(19)	N(1)-C(9)-N(5)	123.7(2)
O(4)-N(8)-C(2)	117.20(19)	N(2)-C(9)-N(1)	123.8(2)
O(1)-N(10)-O(2)	123.93(17)	N(2)-C(9)-N(5)	112.49(17)
O(1)-N(10)-C(4)	116.68(15)		

S6. H and ^{13}C NMR spectra for all new compounds.

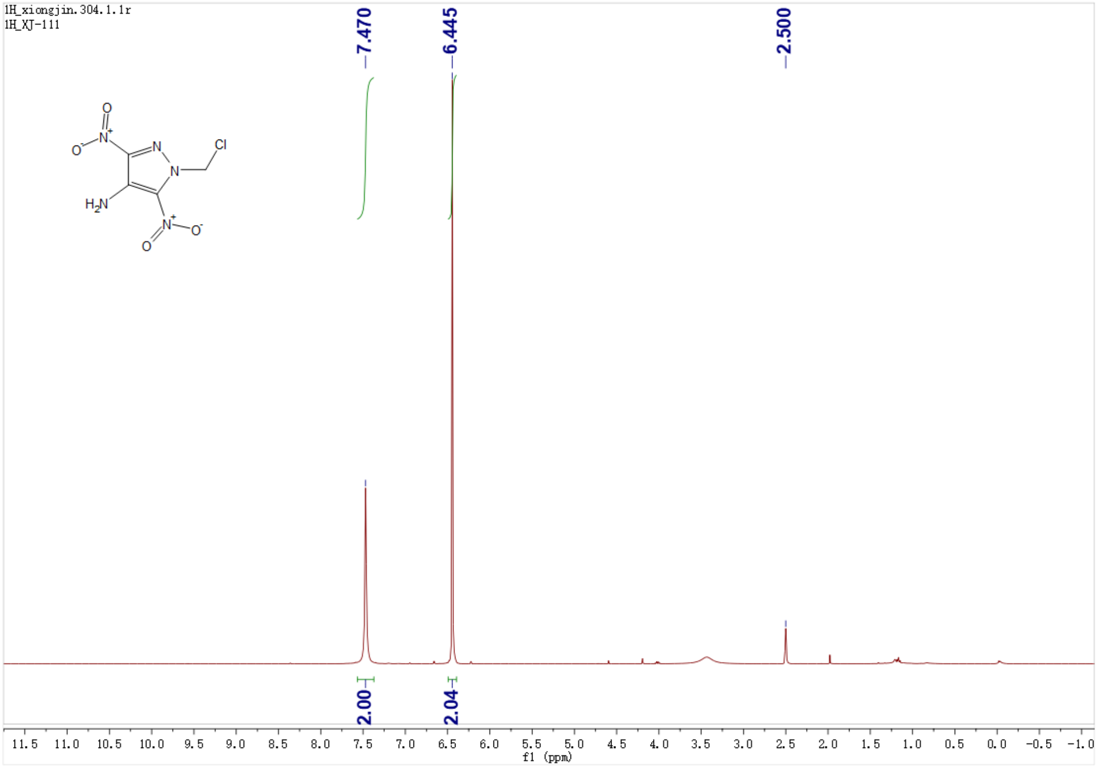

Figure S1. ^{1}H NMR spectrum of **CDPA** in d_6-DMSO.

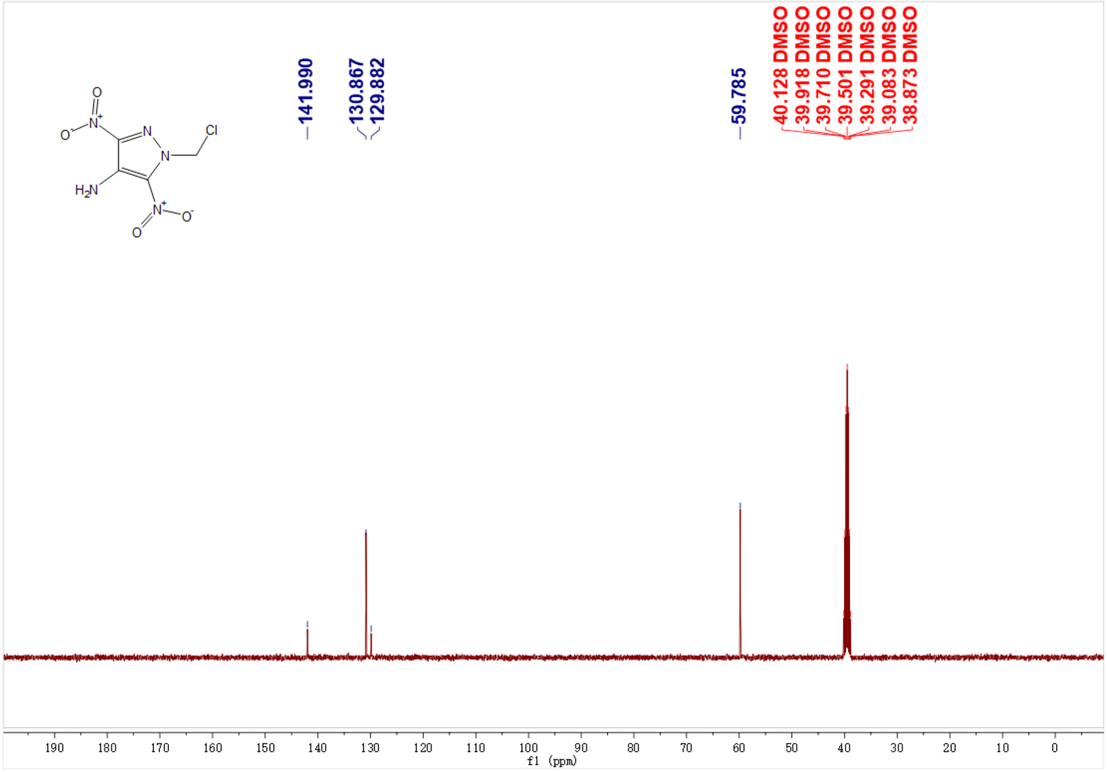

Figure S2. ^{13}C NMR spectrum of **CDPA** in d_6-DMSO.

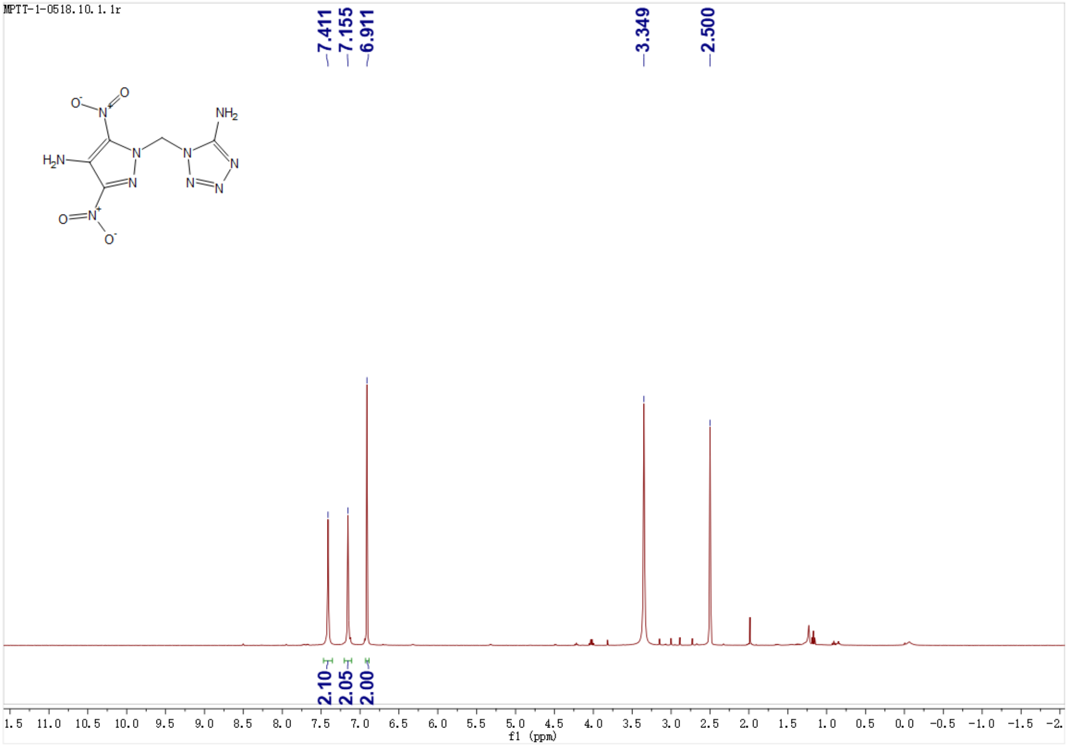

Figure S3. ^{1}H NMR spectrum of **DMPT-1** in d_6-DMSO.

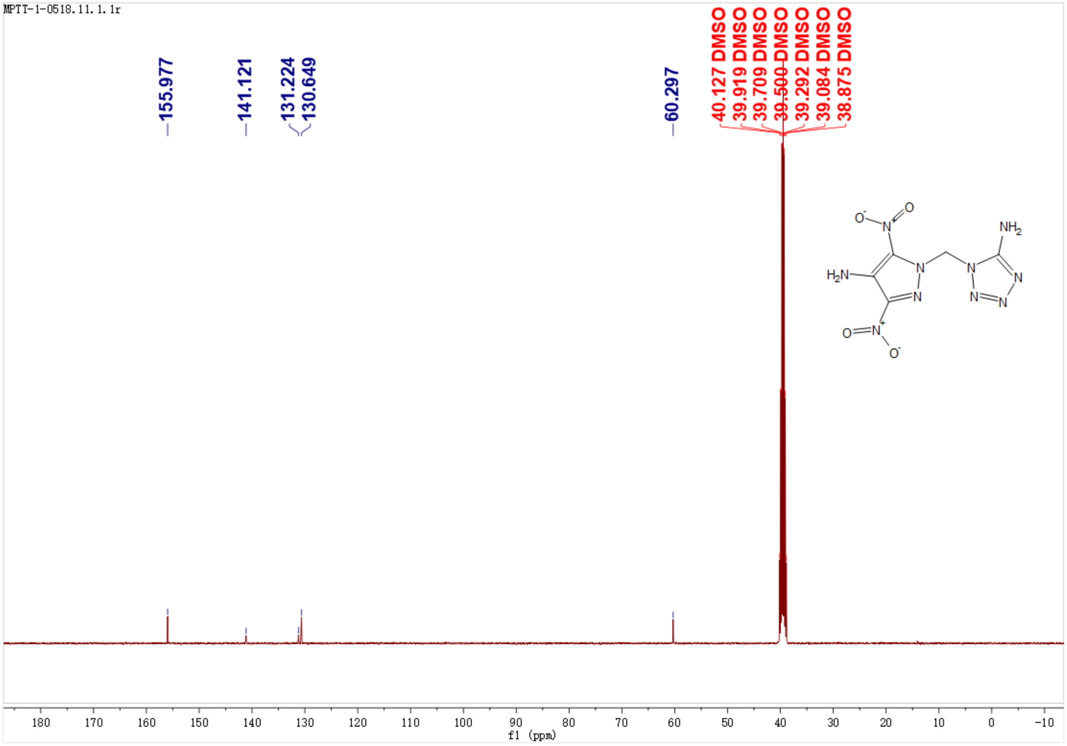

Figure S4. ^{13}C NMR spectrum of **DMPT-1** in d_6-DMSO.

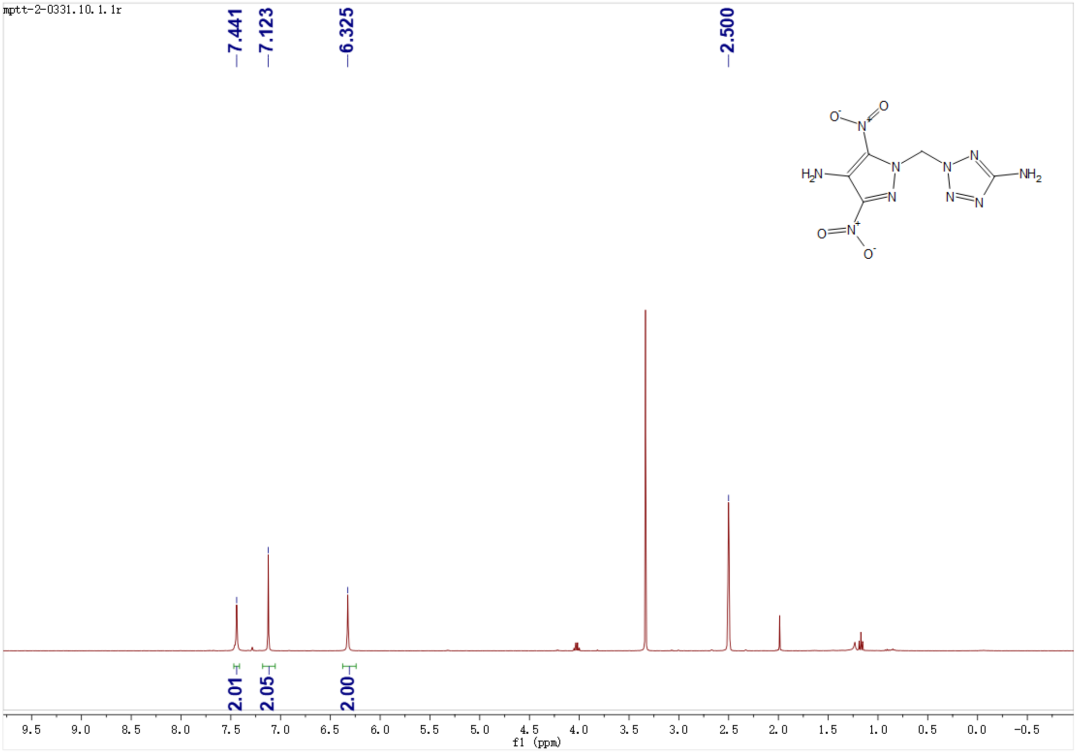

Figure S5. ^{1}H NMR spectrum of **DMPT-2** in d_6-DMSO.

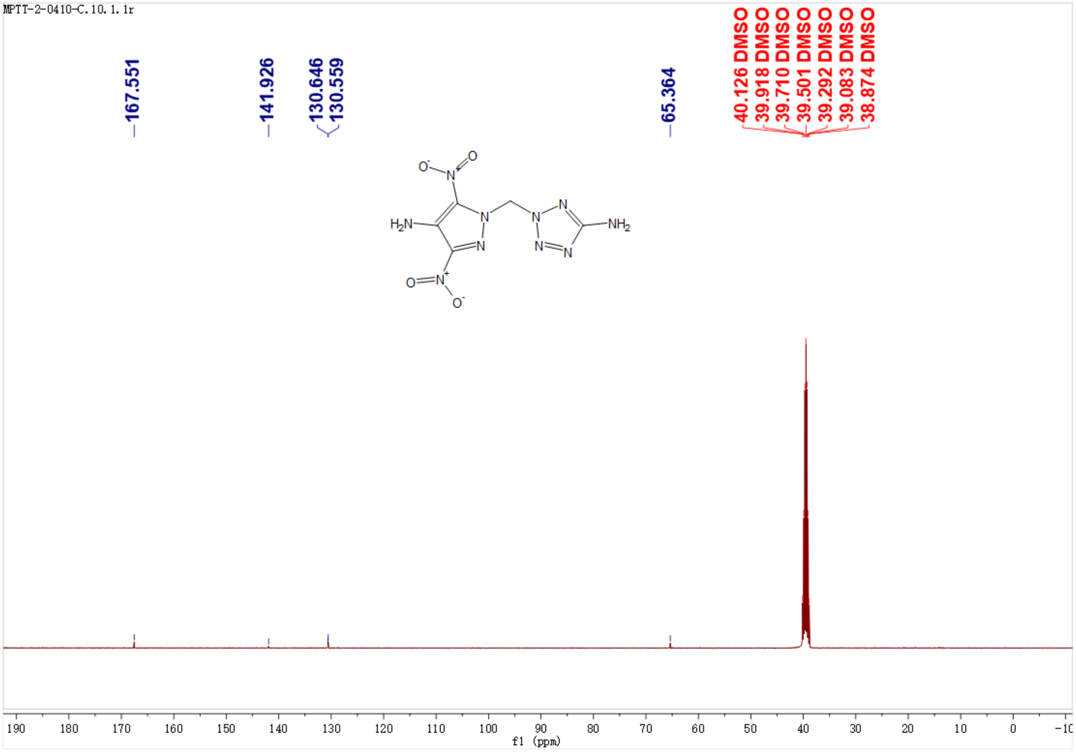

Figure S6. ^{13}C NMR spectrum of **DMPT-2** in d_6-DMSO.

S7. DSC curves of the title compounds

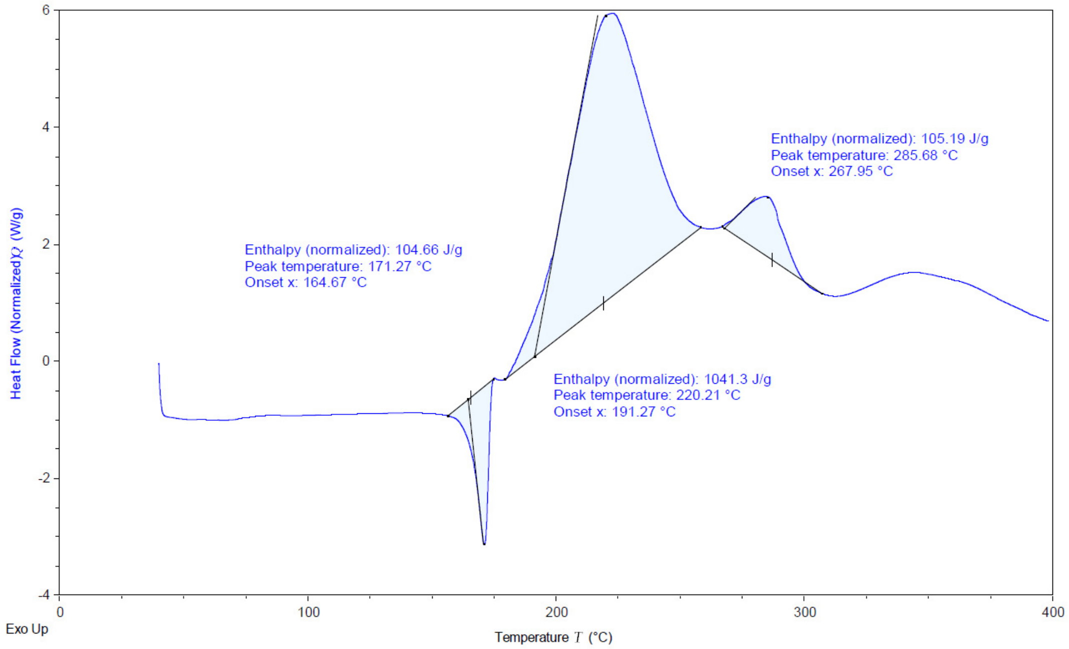

Figure S7. DSC curve of compound **DMPT-1** at 10 °C min^{-1}.

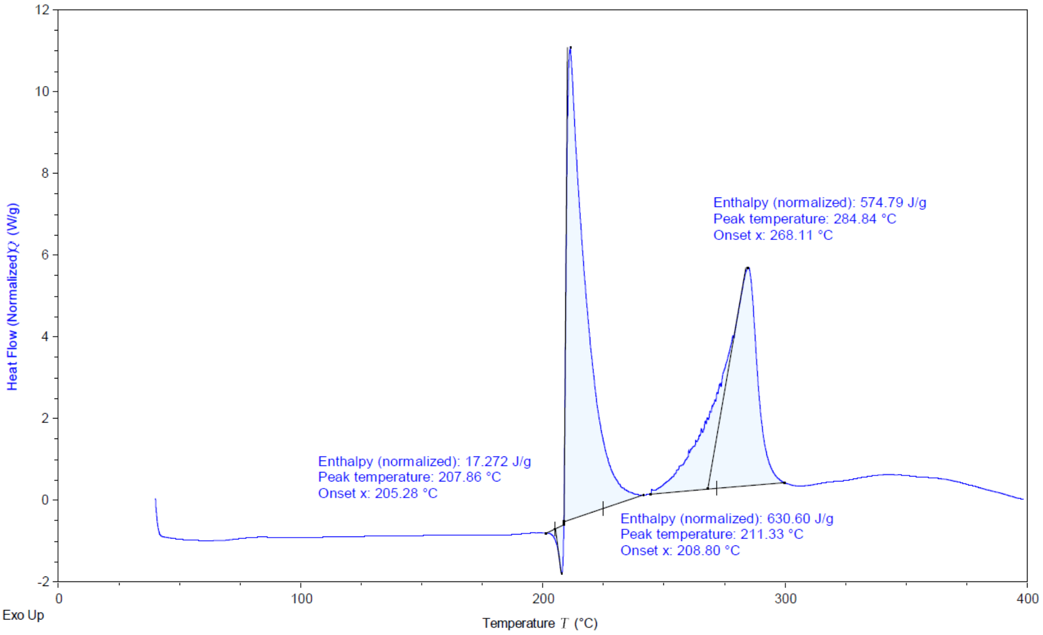

Figure S8. DSC curve of compound **DMPT-2** at10 °C min^{-1}.

References

1. Becke, A. D., Density-functional thermochemistry. III. The role of exact exchange. *J. Phys. Chem.* 1993, **98**, 5648; [CrossRef]
2. Stephens, P. J.; Devlin, F. J.; Chabalowski, C. F.; Frisch, M. J., Ab Initio Calculation of Vibrational Absorption and Circular Dichroism Spectra Using Density Functional Force Fields. *J. Phys. Chem.* 1994, **98**, 11623. [CrossRef]
3. Westwell, M. S.; Searle, M. S.; Williams, D. H., Empirical Correlations between Thermodynamic Properties and Intermolecular Forces. *J. Am. Chem. Soc.* 1995, **117**, 5013. [CrossRef]
